# ASSESSMENT OF A U.S. NURSING STUDENT EXCHANGE PROGRAM FOCUSED ON JAPANESE OLDER ADULTS

**DOI:** 10.1093/geroni/igae098.3795

**Published:** 2024-12-31

**Authors:** Keiko Ono

**Affiliations:** Aomori University of Health and Welfare, Aomori-City, Aomori, Japan

## Abstract

From May 13 to 15, 2024, undergraduate nursing students from the U.S. participated in an international exchange at a university in Japan. A one-day program explicitly focused on learning about services for older adults in Japan: this study aimed to evaluate that program. On May 14, 2024, an intervention program was conducted in Japan, featuring lectures on Long-Term Care Insurance and other issues concerning older adults, including visits to nursing care facilities for older adults. The study involved seven undergraduate American nursing students. A qualitative descriptive research design was employed to assess the program’s impact. Before the intervention, participants individually responded to several questions via QR Codes using Microsoft Forms. These responses served as the basis for a group discussion, during which students summarized their thoughts on a sheet. After the intervention, the group discussed and revised their initial thoughts on the same questions. Finally, participants answered several questions using QR codes. The survey results indicated that none of the students were familiar with services for older Japanese adults before the program. However, after the program, the percentage of students interested in nursing care services for older adults in Japan increased from 57.1% to 71.4%. Additionally, the program positively influenced the student’s perception of older adults. They described older adults with terms such as “sociable,” “active,” “happy,” “willing to live,” and “well cared for.” This program may have made students perceive older adults in Japan as positive and content.

